# Challenges in pharmacovigilance

**DOI:** 10.4103/0253-7613.71882

**Published:** 2010-12

**Authors:** R. K. Dikshit

**Affiliations:** Chief Editor, Indian Journal of Pharmacology, Department of Pharmacology, B.J. Medical College, Ahmedabad - 380 016, India. E-mail: chiefeditorijp@gmail.com

The National Pharmacovigilance Programme of India (NPVI) has recently been launched. A renewed effort, yet, a right step in the right direction, something that is our own, designed, funded, and executed by us. It deserves not only our best wishes, but also a wholehearted cooperation. However, owing to the past experiences, a certain amount of apprehension is difficult to be avoided. In order to help programme incorporate the suitable in-built devices right from the beginning, the challenges must be discussed now at the out-set.

Pharmacovigilance has always been important, as it should have been, although it became visible in India not so long ago. Several factors contribute to its gradually enhancing value. Some of these are (i) a large number of new medicines being introduced regularly, (ii) shortcomings of preclinical testing and premarketing clinical trials, (iii) simultaneous introduction of new medicines globally that disallows us the luxury of the experience gained during “lag period,” (iv) the new patent regime makes it necessary for our pharmaceutical industry to discover and develop the new medicines and (v) the phenomenal growth of “clinical research” in India that exposes our unsuspecting population to the new or less well-known molecules. A large population, a big disease load, easy availability of prescription medicines over the counter and the existence of multiple systems of healthcare (and their possible interplay) add up to enhance the importance of pharmacovigilance. Indeed, it should be considered as a national priority.

There are many prerequisites for a pharmacovigilance programme, and then there are many problems too. Combined together these prerequisites and problems are the real challenges we need to face. *Political will* is first, foremost and an absolute necessity. Government has a key role to play in planning and sustaining a programme. Without a strong political will this cannot be done. Second, the *system* in place should be just right. It should be easily approachable, widely prevalent (in every nook and corner of the country), and commonly known. It should facilitate reporting and ensure that all reports are handled appropriately until a logical conclusion is reached. A system that is *ad-hoc* in nature and is designed to work for a limited period (with limited grants!) will be meaningless. Pharmacovigilance has to be a continuous activity and to be successful we should have a permanent setup that works either from within an official organization or outside. That brings us to another challenge, and that is the nature and extent of involvement of *private sector*, if any, in this activity. Similarly, our *relationship* with other national or international agencies in India or abroad would also require a thoughtful consideration with a view to make it a real collaborative work.

Pharmacovigilance has three major steps, detection, deduction, and decision (including dissemination). Detection, as we all know, is sometimes done through experimental techniques (e.g., randomized clinical trials) but more commonly it employs a number of observational (or pharmacoepidemiological) methods (e.g., spontaneous reports, case series, intensive monitoring, cohort studies, case–control studies, etc). Of all these, the spontaneous reporting seems to be our best bet for many reasons. *Reporting*, however, is an important challenge as we hardly have that culture. We are a tolerant society and even that word “report” sounds unpleasant to us. Other causes for an inadequate reporting can be a busy schedule of healthcare professionals and their greater emphasis on disease (rather than drugs). Suitable training and appropriate education particularly during the formative (undergraduate) years may prepare the prescribing minds in this direction. Pharmacovigilance, unfortunately, is one activity where there are hardly any rewards or incentives. One may debate about them and also for making it to be a statutory requirement. The process of “deduction” is the analysis of available information. This is an important step as merely collecting reports (and not doing anything appropriate to them) is simply like having a number of surveillance cameras with no one to monitor them and take the necessary action when needed. Information analysis is a highly specialized job and methods used may be quite complex. We may wish these to be simple and sure, to be able to provide an answer with greater certainty even when the available data are incomplete. Causality scales are an example where we may like to have an improvement to begin with. Existence of an *efficient system of communication* is an important prerequisite. We should not keep our work confined to certain databases only. Constructive feedbacks, interim analyses, eventual decisions should all be communicated to prescribers (and all concerned) as effectively and as early as possible. This will help us to avoid the undue public alarms and misinformation/misinterpretation at various levels.

Do we have adequate number of properly *trained* persons to take care of all the aspects of pharmacovigilance? I do not think so. A bigger concern is that currently we do not have many such organizations that can impart the quality training in this field.

It is said that *pharmacovigilance is not easy and its errors and problems tend to be repeated*.[1] True, we have seen that before. We have had our share of errors and problems. We should now hope that the new programme will have a smooth sailing. It will help us improve the patient safety significantly.
